# An automated Design-Build-Test-Learn pipeline for enhanced microbial production of fine chemicals

**DOI:** 10.1038/s42003-018-0076-9

**Published:** 2018-06-08

**Authors:** Pablo Carbonell, Adrian J. Jervis, Christopher J. Robinson, Cunyu Yan, Mark Dunstan, Neil Swainston, Maria Vinaixa, Katherine A. Hollywood, Andrew Currin, Nicholas J. W. Rattray, Sandra Taylor, Reynard Spiess, Rehana Sung, Alan R. Williams, Donal Fellows, Natalie J. Stanford, Paul Mulherin, Rosalind Le Feuvre, Perdita Barran, Royston Goodacre, Nicholas J. Turner, Carole Goble, George Guoqiang Chen, Douglas B. Kell, Jason Micklefield, Rainer Breitling, Eriko Takano, Jean-Loup Faulon, Nigel S. Scrutton

**Affiliations:** 1 Manchester Centre for Synthetic Biology of Fine and Speciality Chemicals (SYNBIOCHEM), Manchester Institute of Biotechnology, The University of Manchester, Manchester, M1 7DN UK; 2 School of Computer Science, The University of Manchester, Manchester, M13 9PL UK; 3 School of Chemistry, The University of Manchester, Manchester, M13 9PL UK; 4 School of Life Sciences, Tsinghua University, 100084 Beijing, China; 5 MICALIS, INRA-AgroParisTech, Domaine de Vilvert, 78352 Jouy en Josas Cedex, France

## Abstract

The microbial production of fine chemicals provides a promising biosustainable manufacturing solution that has led to the successful production of a growing catalog of natural products and high-value chemicals. However, development at industrial levels has been hindered by the large resource investments required. Here we present an integrated Design–Build-Test–Learn (DBTL) pipeline for the discovery and optimization of biosynthetic pathways, which is designed to be compound agnostic and automated throughout. We initially applied the pipeline for the production of the flavonoid (2*S*)-pinocembrin in *Escherichia coli*, to demonstrate rapid iterative DBTL cycling with automation at every stage. In this case, application of two DBTL cycles successfully established a production pathway improved by 500-fold, with competitive titers up to 88 mg L^−1^. The further application of the pipeline to optimize an alkaloids pathway demonstrates how it could facilitate the rapid optimization of microbial strains for production of any chemical compound of interest.

## Abstract (web summary)

Pablo Carbonell et al. present an automated pipeline for the discovery and optimization of biosynthetic pathways for microbial production of fine chemicals. They apply their pipeline to the production of the flavonoid (2S)-pinocembrin in *Escherichia coli* and show improvement of the pathway by 500-fold.

## Introduction

Recent technical advances in synthetic biology, including rapid DNA assembly^1^, genome editing^2^, comprehensive pathway refactoring^3^, high-throughput screening^4^, and powerful pathway design tools^5^, are enabling the increased automation of microbial chemical production processes^6,7^. Academic and industrial biofoundries are increasingly adopting an engineering approach based on the iterative application of the DBTL cycle that has long been a central element of product development in traditional engineering disciplines^8^. Here we present an automated DBTL pipeline for the rapid prototyping and optimization of biochemical pathways in microbial chassis, which integrates a unique combination of these new technologies. The pipeline is designed to be agnostic regarding the target compound and runs from the in silico selection of candidate enzymes, through automated parts design, statistically guided and robot-assisted pathway assembly, rapid testing and rationalized redesign, providing an iterative DBTL cycle underpinned by computational and laboratory automation. This is a major step forward toward automating the DBTL cycle to develop biomanufacturing solutions for industrial chemical production.

## Results

### The automated Design–Build-Test–Learn pipeline

The Design stage of the pipeline includes an integrated suite of novel software tools. For any given target compound, tools for automated pathway and enzyme selections are RetroPath^9^ and Selenzyme^10^ (http://selenzyme.synbiochem.co.uk), respectively. Reusable DNA parts are then designed with the simultaneous optimization of bespoke ribosome-binding sites and enzyme coding regions using the in-house-developed PartsGenie software^11^ (https://parts.synbiochem.co.uk). Genes and regulatory parts are combined in silico into large combinatorial libraries of pathway designs, which are statistically reduced using design of experiments (DoE) to smaller representative libraries. Such libraries allow the efficient exploration of the design space resulting in tractable numbers of samples for laboratory construction^12,13^ and screening^14,15^, alleviating the need for high-throughput systems. Our publicly available custom software (https://parts.synbiochem.co.uk/plasmidGenie) then produces assembly recipes and robotics worklists to enable automated ligase cycling reaction^16^ for pathway assembly (Supplementary Data 1 and 2), and all DNA part designs and plasmid assemblies are simultaneously deposited in a JBEI-ICE repository^17,18^, providing unique IDs for sample tracking. The Build stage begins with commercial DNA synthesis (Supplementary Tables 1 and 2), then part preparation via PCR, followed by reaction setup for pathway assembly by ligase cycling reaction on robotics platforms. After transformation in *E. coli*, candidate plasmid clones (Supplementary Tables 3 and 4) are quality checked by high-throughput automated purification, restriction digest and analysis by capillary electrophoresis, followed by sequence verification. To Test, constructs are introduced into selected production chassis and automated 96 multi-well growth/induction protocols run (Supplementary Fig. 1). The detection of target product and key intermediates from cultures begins with automated extraction followed by quantitative screening involving fast ultra-performance liquid chromatography coupled to tandem mass spectrometry with high mass resolution. The data extraction and processing are based on custom-developed and open-source R scripts. The Learn stage involves identifying the relationships between observed production levels and design factors through the application of statistical methods and machine learning, and this includes suitable statistical validation.

**Fig. 1:**
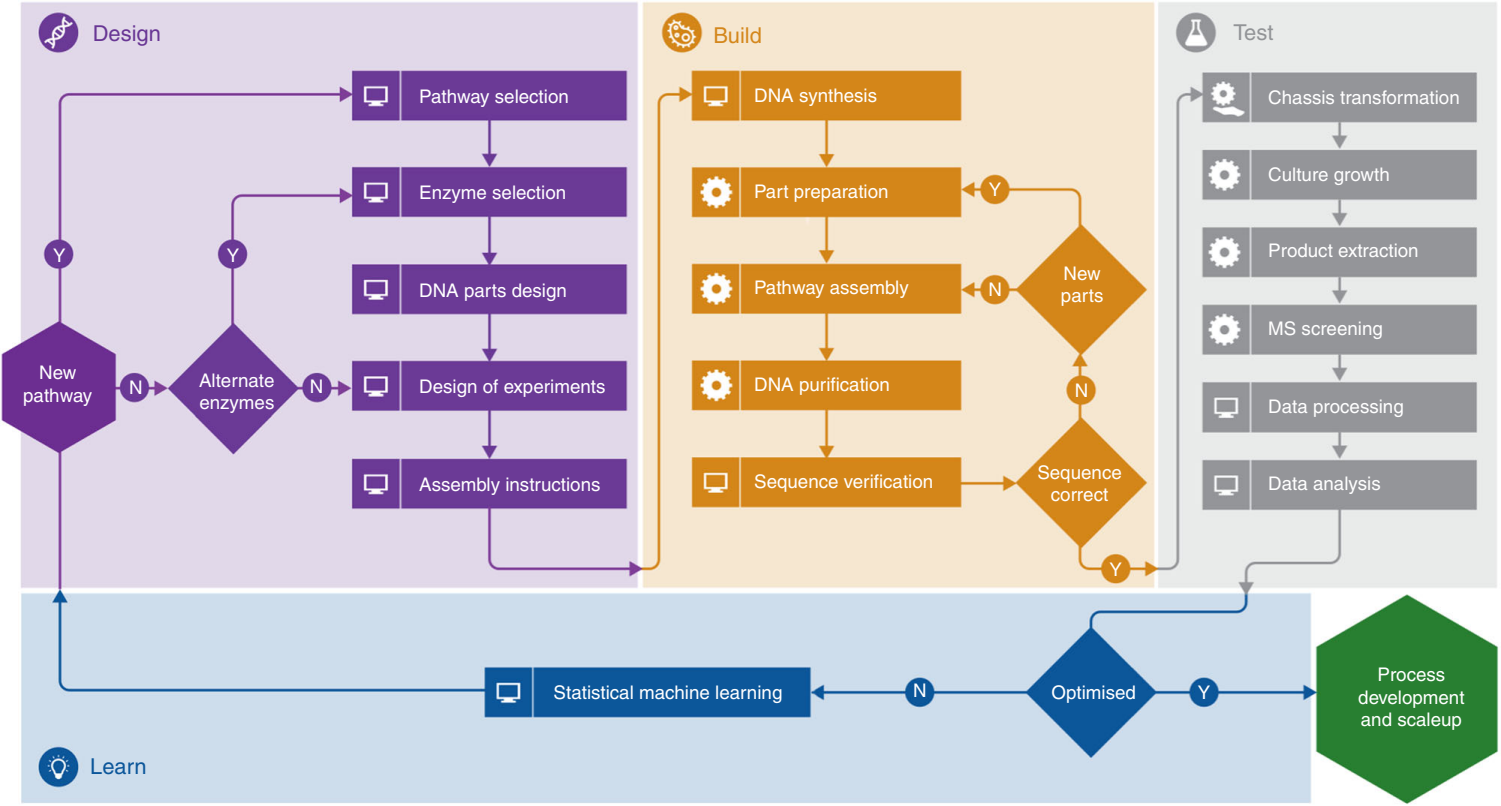
The SYNBIOCHEM Design/Build/Test/Learn pipeline for microbial production of fine and speciality chemicals. The pipeline starts at the Design stage (purple) with pathway (RetroPath) and enzyme (Selenzyme) selection tools. Selected DNA parts are sequence optimized (PartsGenie), combined into plasmid libraries through design of experiments (SBC-DoE), and automated assembly instructions are generated (DominoGenie). The Build stage (orange-yellow) prepares assembly parts from commercially synthesized DNA, and assembles them into plasmids via ligase cycling reaction, according to automatically generated worklists driving laboratory automation. Assembled plasmids are first checked by high-throughput restriction digest analysis using capillary electrophoresis, then by commercial Sanger-based sequencing. The Test stage (gray) encompasses high-throughput methods for the growth of microbial production cultures, automated product extraction, and screening via fast-liquid chromatography QqQ mass spectrometry. Data are processed and analyzed with open-source R scripts. Results are analyzed at the Learn stage (blue) through predictive models using statistical methods and machine learning to inform the next round of design. After a number of iterations of this DBTL cycle, successful prototypes are taken forward to process development and scale-up

The pipeline is built with the aim of automating each identified bottleneck in the DBTL cycle. In that way, the pipeline provides an efficient and streamlined approach to pathway engineering. Automation is a key to achieving this goal. As shown in Fig. 1, most of the steps in the pipeline have been replaced by automated workflows. Currently, some manual interventions remain in this workflow, in particular PCR clean-up and host–cell transformation are carried out off deck, and plates need to be manually transferred between certain platforms. However, there is no reason why these steps could not also be automated in the future. The pipeline is designed in a modular fashion which would allow other laboratories to replace individual pieces of equipment or protocols to adopt their own methods. The principles and processes of the pipeline would be preserved in this manner. This also allows future-proved flexibility as technology develops in the future. Our suite of design tools can be easily deployed in other biofoundries. All designed parts and plasmids are deposited from a centralized ICE repository providing the transition from Design to Build. Automated worklist generation for ligase cycling reaction assembly can facilitate standardizing the Build step. The data tracking systems underpin the transition from Build to Test and Learn. Efforts from several groups on standardization of the SOPs/software protocols to provide common platforms and transferability are therefore enabling technologies for the integration as far as they remain open source. As they become community standards, such standard protocols will be incorporated into our pipeline. The aim of the pipeline is to perform rapid prototyping in order to identify the best combination of genetic parts leading to high producer strains. Once top producers have been identified, a second stage is pathway optimization followed by integration of pathways into the organism’s genome for scale-up. The pipeline infrastructure and its associated methodologies can be easily adapted for use with a wide range of industrial microorganisms, not just *E. coli*. There are certain sequence design parameters, such as regulatory elements, codon optimization and experimental methods (e.g., transformation and growth conditions), that may require modification to accommodate the requirements of different microbes, but the modular nature of the pipeline streamlines the introduction of species-specific constraints without disrupting the overall workflow and iterative nature of the automated DBTL approach.

### Application of the pipeline to flavonoids production

**Fig. 2:**
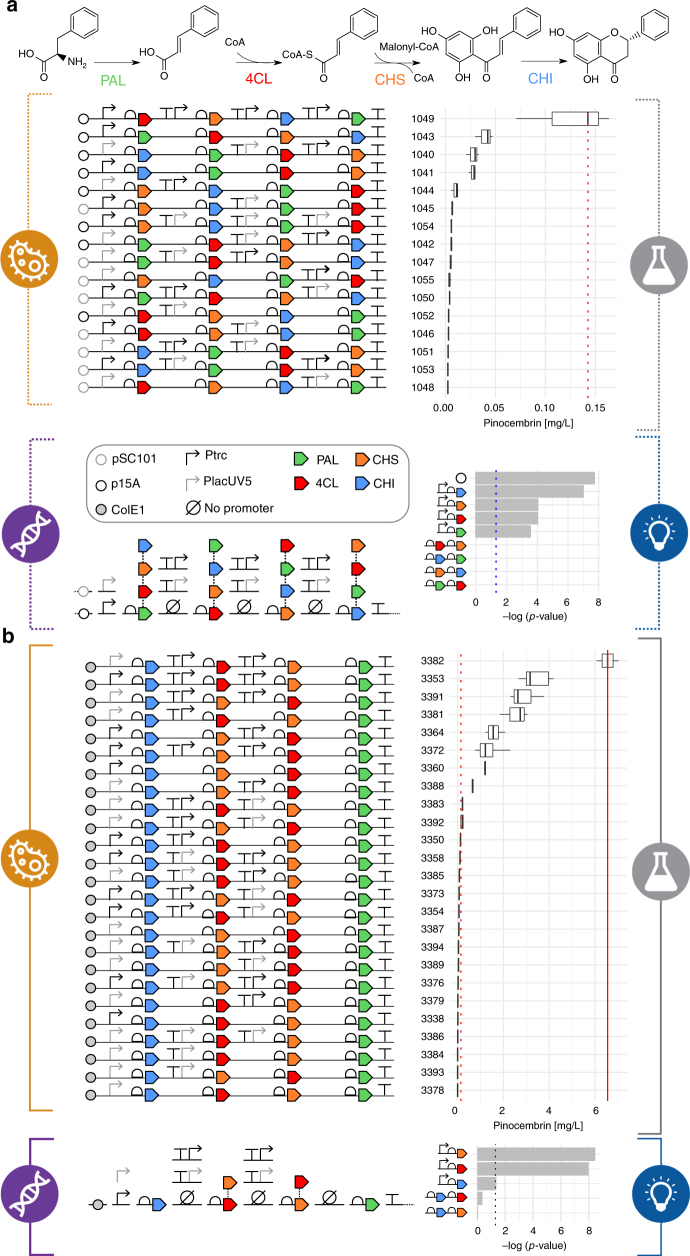
Combinatorial optimization of the (2S)-pinocembrin pathway through the Design/Build/Test/Learn cycle. **a** A biosynthetic pathway composed of four enzymes (PAL, 4CL, CHS, and CHI; see Supplementary Table 2) was initially selected^21^. In the first DBTL cycle, a combinatorial library totaling 2592 pathway configurations was designed by varying the order of pathway genes, promoter parts (P_trc_ and P_lacUV5_), and plasmid copy numbers (pSC101 and p15a). Through the application of statistical DoE, the designed library was reduced to 16 representative constructs. This pathway library was assembled and expressed in *E. coli* DH5α to test pinocembrin titers. Statistical analysis was then used to assess the relative effects of the different design factors tested. **b** In the second DBTL cycle, a new focused combinatorial library was designed, based on experimental factors from the first cycle which correlated with pinocembrin titer. For this second full-factorial library, PAL was fixed at the end of the pathway and CHI at the beginning, while CHS and 4CL were allowed to exchange positions with or without promoter parts

Our first application of the pipeline targeted flavonoid production pathways expressed in *E. coli*. Flavonoids are among the most structurally diverse classes of natural products^19^, and pinocembrin serves as a key precursor to this diversity^20^. Automated enzyme selection for the pinocembrin pathway had already been validated using our RetroPath^21^ software and served as a compatible start point for the rest of the pipeline. The four selected enzymes (phenylalanine ammonia-lyase (PAL), chalcone synthase (CHS) and chalcone isomerase (CHI) (all from *Arabidopsis thaliana*) and 4-coumarate:CoA ligase (4CL) (from *Streptomyces coelicolor*, strain ATCC BAA-471/A3(2)/M145)) convert l-phenylalanine to (2*S*)-pinocembrin with the requirement for malonyl-CoA (Fig. 2).

In order to efficiently explore the design space, an initial library covering a wide range of variants was designed with the following parameters: four levels of expression by vector backbone selection; varying the copy number from medium (p15a origin) to low (pSC101 origin) and including a strong (P_trc_) or a weak promoter (P_lacUV5_)^22^. Further regulation was introduced by considering each intergenic region in the pathway to include a strong, weak, or no promoter. Finally, another factor was introduced by varying the position of each of the four genes, resulting in 24 permutations (Fig. 2a, Design). This combinatorial design approach produced 2592 possible configurations (4 × 3 × 3 × 3 × 24).

DoE based on orthogonal arrays combined with a Latin square for positional arrangement of the genes was applied to reduce the 2592 combinations down to 16 representative constructs, achieving a compression ratio of 162:1. All 16 constructs in this reduced library were successfully assembled (Fig. 2a, Build) and sequenced-verified. The library was screened for production of pinocembrin and the pathway intermediate cinnamic acid in *E. coli* DH5α using an HTP 96-Deepwell plate-based growth pipeline with media and culture conditions approximated to that of Fehér et al.^21^ This initial library was found to produce pinocembrin titers ranging from 0.002 to 0.14 mg L^−1^ (Fig. 2a, Test and Supplementary Data 3). Measured pinocembrin titers for the 16 constructs were statistically analyzed to identify the main factors influencing production from the design parameters (Fig. 2a, Learn). Vector copy number had the strongest significant effect on pinocembrin levels (*P* value = 2.00 × 10^−8^), followed by a positive effect of the CHI promoter strength (*P* value = 1.07 × 10^−7^). Weaker effects were observed for CHS (*P* value = 1.01 × 10^−4^), 4CL (*P* value = 1.01 × 10^−4^), and PAL (*P* value = 3.06 × 10^−4^) promoter strengths, respectively. Every construct also produced high levels of the intermediate cinnamic acid relative to pinocembrin, suggesting that PAL enzyme activity is high even at low levels of expression. The effects of relative gene order on pinocembrin production were not significant.

The design specifications used in the second round were focused on some specific region of the design space based on existing knowledge from the first round. Design constraints were defined as follows: (a) a high copy number origin of replication (ColE1) was selected for all constructs, because the statistical analysis showed that vector copy number was the strongest ordinal factor observed with a positive effect on production titers; (b) CHI, the expression of which was identified as having a strong effect on pinocembrin production, was kept at the beginning of the pathway to ensure it was always directly downstream of a promoter; (c) a lesser effect of 4CL and CHS expression levels was also identified and so these genes were allowed to exchange positions in the middle of the construct with the inclusion of no, low (P_lacUV5_), or high (P_trc_) strength promoters in front of each; (d) high levels of cinnamic acid, the product of PAL activity, were observed from all constructs (Supplementary Data 3), suggesting that expression levels of the gene were not limiting. Therefore, the location of the PAL gene was kept fixed at the 3′ end of the assembly as the last gene within a 2–4 gene operon. Even though PAL promoter strength had some impact on pinocembrin titers according to the statistical analysis, its effect was smaller than for the other promoters and therefore was not considered a significant bottleneck because of the observed cinnamic acid accumulation. In addition, CHI and PAL were kept at the first and last positions for practical reasons in order to focus the test on exchanging promoters on 4CL and CHS at the middle section of the construct.

The resulting library design for the second round of testing consisted of just 36 constructs (2 × 3 × 3 × 2) and so the full-factorial design was selected for assembly (Fig. 2b, Design). The first attempt at constructing this library yielded 25 of 36 constructs with verified sequences (Fig. 2b, Build). Among the 25 successfully built constructs, 19 (76%) showed median titers of pinocembrin above the highest producer in first round (Supplementary Data 4), with ten designs (40%) showing a marked increase in pinocembrin production of up to 46-fold. The best producer (pathway 3382) yielded a median level of 6.6 mg L^−1^ pinocembrin (Fig. 2b, Test and Supplementary Data 4). Statistical analysis of this library’s performance revealed that the most decisive factor was the promoter strength in front of CHS (*P* value = 3.46e × 10^−9^), followed by the strength effect of the 4CL promoter (*P* value = 1.03 × 10^−8^). Finally, the effect of the CHI promoter was of lower significance (*P* value = 3.34 × 10^−2^), most likely because contrary to 4CL and CHS, a promoter was kept in front of CHI in all constructs. The effect of exchanging CHS and 4CL positions was not found significant, confirming the results already obtained in iteration 1. Pathway 3382 already had the strongest promoter (P_trc_) upstream of the genes for CHS and 4CL and there were no other pathway permutations in the library predicted to improve titers although stronger promoter parts could be designed for future pathway assemblies. This improvement of pathway performance in the second round over the first confirmed the importance of the design rules obtained from the Learn stage.

**Fig. 3:**
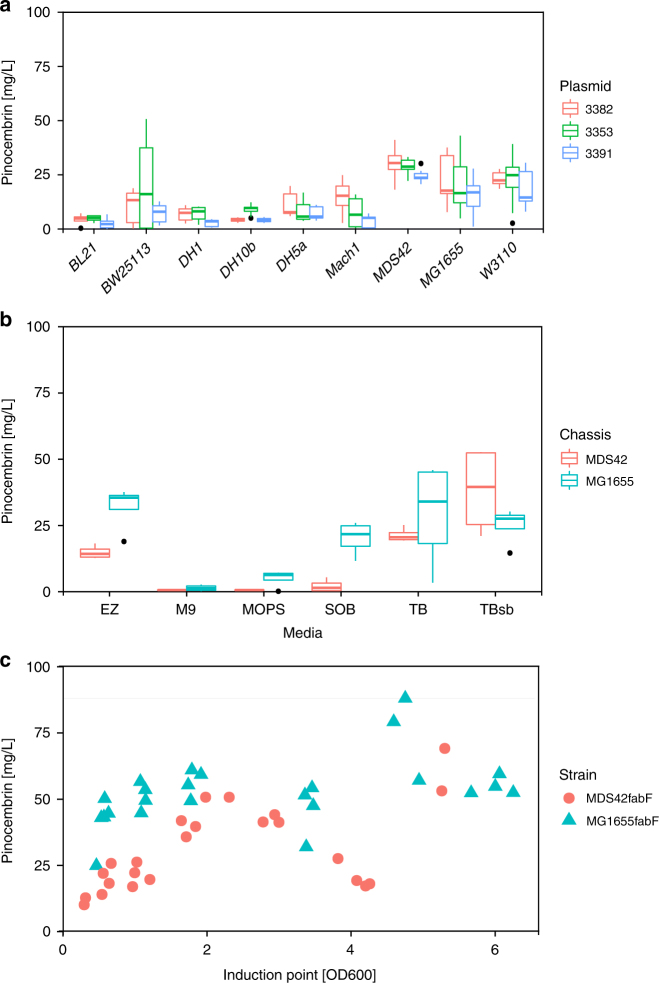
Process development and optimization of (2S)-pinocembrin production. **a** Chassis selection was performed by expressing the best-performing constructs from the second DBTL cycle (3382, 3353, and 3391; see Supplementary Table 1) in nine different *E. coli* strains (listed in Table 1) and the results are displayed as box-whisker plots, indicating median and interquartile range. **b** Media screening was performed by expressing the best construct (3382) in the two best chassis (MG1655 and MDS42) and monitoring pinocembrin in six different growth media (listed in Table 2). **c** Further optimization was investigated by screening *fabF::kan* mutants of the best chassis (MG1655 and MDS42) transformed with the best construct (3382), with pathway induction at different culture densities (OD_600_)

Other studies have employed additional strategies for boosting titers in the flavonoids pathway, including chassis engineering to increase malonyl-CoA^21,23,24^ or phenylalanine^24^ availability, resulting in titers of up to 40 mg L^−1^ ^21,23,24^; and regulation of growth through substrate feeding (glucose and phenylalanine) and pH regulation in fermenters, resulting in the highest reported titer of 68 mg L^−1^ ^23^. In order to assess the production capabilities of the prototype selected by the pipeline, we performed additional screening for optimal chassis, growth media, and strain. The three best-performing pathways from the second library (3382, 3353, and 3391) were screened in triplicate in nine different *E. coli* strains, including K-12, B, and W strains derivatives (Fig. 3a and Supplementary Data 5). A dramatic range in pinocembrin production titers was observed (from 0.1 to 34.7 mg L^−1^), with K-12 strains displaying the highest titers. Pathway 3382 was the highest producer across the top-performing strains, consistent with the library screens conducted in DH5α. The MDS42 strain with pathway 3382 produced the highest median titer of 30.4 mg L^−1^, representing a five fold increase with respect to the titer obtained in DH5α. Some strains displayed large titer variation and individual clone titers were observed up to 50.7 mg L^−1^ (BW25113 with 3353). Media screening was performed for the best-performing pathway (3382) in seven different growth media (Fig. 3b and Supplementary Data 6). The MDS42 strain with pathway 3382 produced a mean titer of 38 mg L^−1^ in TBsb media. The MG1655 strain with pathway 3382 produced the best mean titers of 32 mg L^−1^ of pinocembrin in EZ media, however, the highest individual clone titers were observed in phosphate-buffered TB media. Finally, we adopted the well-documented strategy to improve flavonoid production by reducing malonyl-CoA consumption in fatty acid biosynthesis. One approach is to knockout the *fabF* gene encoding the 3-oxoacyl-(acyl carrier protein [ACP]) synthase II enzyme, which incorporates malonyl-CoA into fatty acid biosynthesis^24^. Knockout of the *fabF* gene was performed (by kanamycin resistance cassette insertion) in both MG1655 and MDS42 and then pathway 3382 was introduced. The strains were grown in TB media and pinocembrin production was induced at different culture densities (OD_600_) as shown in Fig. 3c and Supplementary Data 7. Under these conditions, we observed final pinocembrin of up to 88 mg L^−1^ for the MG1655 *fabF::kan* strain with pathway 3382, higher than those reported in the literature.

### Application of the pipeline to alkaloids production

**Fig. 4:**
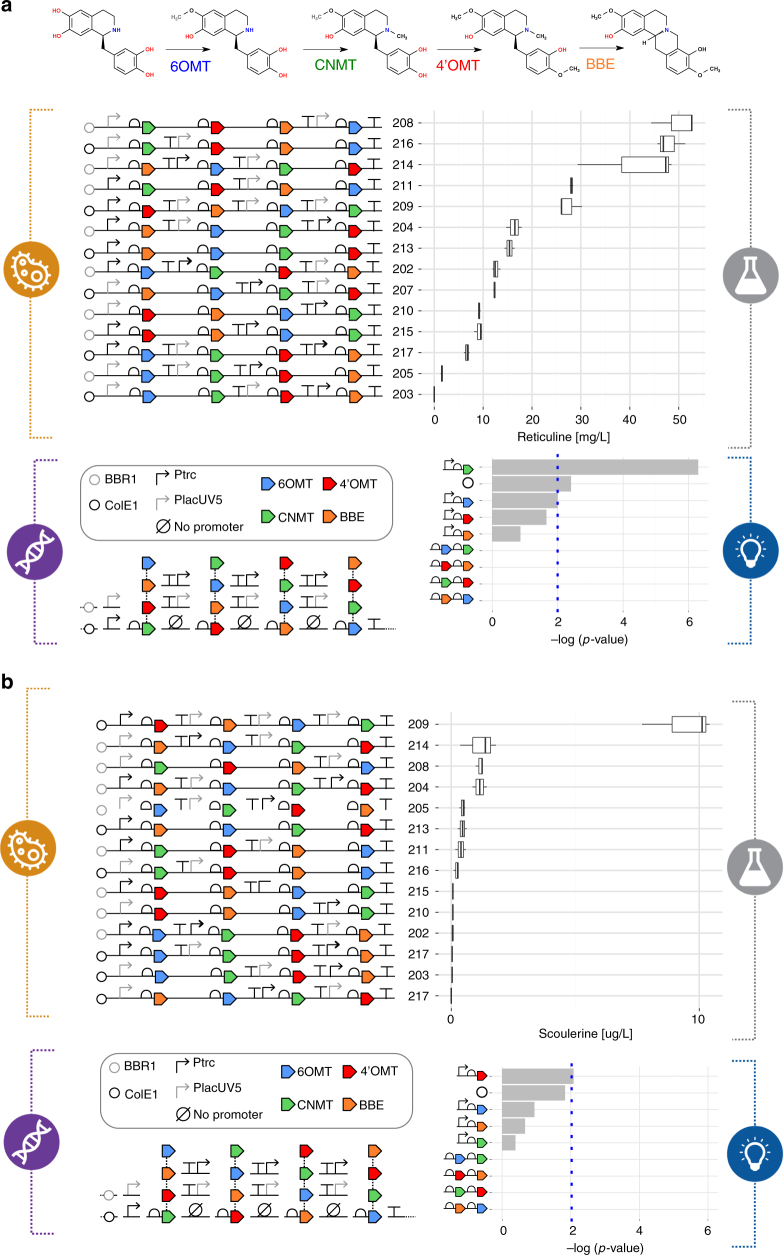
Combinatorial optimization of the (*S*)-reticuline/(*S*)-scoulerine pathway through the Design/Build/Test/Learn cycle. **a** A biosynthetic pathway composed of four enzymes from *Coptis japonica* (6OMT, CNMT, 4′OMT, and BBE; see Supplementary Table 10) was selected. For this DBTL cycle, a combinatorial library totaling 2592 pathway configurations was designed by varying the order of pathway genes, promoter parts (P_trc_ and P_lacUV5_) and plasmid copy numbers (pBBR1 and ColE1 origins). Through the application of statistical DoE, the designed library was reduced to 16 representative constructs, of which 14 were successfully assembled and tested. This pathway library was expressed in *E. coli* DH5α and reticuline titers were quantified. Statistical analysis was then used to assess the relative effects of the different design factors tested. **b** Quantification of scoulerine titers observed for the same 14 constructs and statistical analysis of the relative effects of the different design factors

To demonstrate the applicability of the pipeline to target production of diverse chemical compounds, we used our methods to optimize the expression of an alkaloids pathway in *E. coli*. A three-enzyme pathway transforms (*S*)-tetrahydropapaveroline (THP) into (*S*)-reticuline, the branch point precursor to a wide range of valuable benzylisoquinoline alkaloids, which can be converted by a fourth enzyme into the protoberberine target (*S*)-scoulerine (Fig. 4 and Supplementary Table 4). This example showcases how the pipeline can be modularly applied to optimize sections within longer pathways, in our case the transformation of THP into (*S*)-reticuline and (*S*)-scoulerine in the alkaloids pathway, provided suitable standards for intermediates are available. We applied our statistical DoE approach to reduce a full-factorial construct library of 2592 configurations down to 16 representative constructs. We observed difficulties in assembling the 4′OMT-Ptrc-BBE combination within our constructs, resulting in deletion of either the promoter or part of the BBE gene, suggesting that this arrangement is either unstable or is negatively selected against in the cloning host. As a result, constructs 206 and 212 could not be assembled, and although construct 203 had the correct sequence we failed to detect either reticuline or scoulerine in the production strain. The remaining 13 constructs all produced reticuline over a range of titers up to 50 mg L^−1^ (Fig. 4a and Supplementary Data 2), equal to the best titer reported in the literature for an equivalent three-enzyme pathway in *E. coli*^25^. The Learn stage of our pipeline informed us, through statistical analysis of design factors, that tuning of the promoter strength for the CNMT gene had the most significant effect on reticuline titers (*P* value = 5.12 × 10^−7^). Further conversion of (*S*)-reticuline to (*S*)-scoulerine was modest in our screened library, with a highest titer of just 10 µg L^−1^ (Fig. 4b and Supplementary Data 8). We suspect that this is due to solubility issues with BBE, which is expressed in plants as a transmembrane protein with several *N*-glycosylation modifications. Nevertheless, this alkaloid has not been produced in *E. coli* previously and work is ongoing to improve the activity of BBE in this chassis.

## Discussion

We have successfully implemented an automated DBTL pipeline to streamline the process of microbial engineering for chemical production. The pipeline integrates and combines tools of the Design–Build–Test–Learn cycle of metabolic engineering, providing relatively simple robust protocols. Crucially the pipeline is designed with the potential of operating in a target agnostic manner, applicable to any feasible chemical target. To develop and demonstrate its capabilities, the pipeline was applied to optimize the production of the flavonoid (2*S*)-pinocembrin and the alkaloid (*S*)-reticuline in *E. coli*. By considering relatively few design parameters, large numbers of pathway variants were designed and we demonstrated how using statistical sampling of the initial design space, coupled with automated laboratory protocols for pathway assembly and testing, allowed rapid prototyping in vivo. Both pathways were optimized to produce >50 mg L^−1^ of target, making them competitive with current state-of-the-art titers. For pinocembrin, identification of the key design factors influencing final production titers contributed to a second DBTL round using standardized statistical protocols, which resulted in a further 40-fold improvement in pinocembrin production. The titers observed from the two libraries ranged over three orders of magnitude (Fig. 2), clearly demonstrating the importance of pathway design and optimization. Testing such a large design space is only feasible using this kind of approach, combining intelligent sampling and automated workflows.

The efficiency of the automated pipeline is clearly demonstrated, as it led to a 500-fold increase in titers for the flavonoids pathway with competitive titers up to 88 mg L^−1^, from the screening of just 65 (16 + 25 + 24) variants out of 23,328 (2592 × 9) possible designs. Furthermore, for the alkaloids pathway screening of just 14 variants out of 2592 possible designs identified pathways with reticuline titers equivalent to the current state of the art. Our present pipeline can achieve a full iteration in 2 months, including gene synthesis (typically 1 month) and sequencing (3 days). A second iteration involving parts that have been already ordered could be then accomplished in 3 weeks. These results outperform previously reported DBTL approaches^12,13^ in terms of fold increase in titers compared with number of screened variants. As the application of these strategies becomes more widespread, it is anticipated that DBTL pipeline methodologies for engineering biology will provide new faster, highly predictable and sustainable routes to valuable chemical diversity. We envision deriving common design rules and applying state-of-the-art machine-learning techniques in future cases involving larger data sets and design spaces. Similarly, propagation of design rules will be implemented as part of the pipeline through a workflow that will translate inferred design rules between factors into design constraints for the redesign of the next iteration. The workflow could also be extended beyond part selection to other factors like enzymes, alternative pathways, process conditions, etc.–e.g., finding alternatives to overcome identified bottlenecks. Designed to be applicable to any target compound, the pipeline is intended to be compatible for automation and therefore able to work without any prior knowledge of successful strategies. Our application of an automated DBTL pipeline demonstrates how these strategies can efficiently lead to the discovery and rapid optimization of high-performance pathways, providing the tools to enable a new era in automated agile biomanufacturing.

## Methods

### Design

The pipeline uses the retrosynthesis workflow of RetroPath^9^, and the enzyme sequence selection tool Selenzyme^10^ (http://selenzyme.synbiochem.co.uk) to mine available biochemical knowledge^26^ and allow the initial selection of production pathway(s) through the application of reaction rules-based retrosynthesis; and of enzymes based on biochemical reaction similarity at each transformation step in the pathway. Initial synthetic pathways for pinocembrin were selected based on a RetroPath design^21^; genes for reticuline/scoulerine biosynthesis were selected from *Coptis japonica*^27^. Plasmids were built from individual parts, comprising vector backbones (BglBrick vectors^22^), terminator–promoter pairings and enzyme coding genes with optimized ribosome-binding (RBS) sites. Gene parts were designed with the PartsGenie web application^11^ (http://parts.synbiochem.co.uk). RBS translation initiation rates were set to 15,000^28^, CDS were codon optimized for expression in *E. coli*, and 5′ and 3′ sequences were optimized for assembly via the ligase cycling reaction^16^. Parts were also designed to be compatible for BglBrick^22^ and Golden Gate assembly^29^. All part and plasmid designs were formatted in the Synthetic Biology Open Language (SBOL) version 1^30^ and are available from our ICE repository (https://ice.synbiochem.co.uk)^17,18^.

A combinatorial approach was utilized in designing synthetic plasmids expressing each pathway, using a formalized design of experiments (DoE) method. Our design strategy tried to minimize the number of samples by selecting sets of optimal combinatorial libraries to explore the design space determined by the target chemical–producer pathway to be imported into a chassis organism. The reduced design space through intelligent sampling makes possible adding some level of redundancy at the design stage to the libraries to account for assembly failures. A pathway construct template was initially defined by the sequential composition of plasmid and genetic parts available in the centralized ICE repository. For DoE, each part was considered as a factor and the number of levels associated with the factor was given by the number of possible variations of the design parameter. For the first round of design, in order to reduce the total number of promoters, it was considered as a four-level factor, with two of the levels corresponding to a no-promoter state and the other two to each promoter, respectively. We used two DoE approaches: (a) regular fractional factorial design by means of the planor R package^31^; (b) orthogonal arrays, which are a generalized form of mutually orthogonal Latin squares, by means of the DoE.base R package^32^. We considered an additional factor given by the variation of the positional order of genes. This factor can be used in order to perform permutations of the desired *n* genes within the construct and combinations can be reduced to *n* by using a Latin square. Through the application of orthogonal array design, the initial design round generated 16 variant plasmids, in which the plasmid copy number, the order of enzymes within the plasmid and the strength of promoters were varied.

Each resulting construct in the library was processed through design and optimization algorithms for DNA parts (RBS and CDSs) and plasmids, that were tailored to the chosen assembly method. Ligase cycling reaction was used to assemble plasmids, for which bridging oligos were designed with in-house software, assuming a ligase cycling reaction melting temperature of 70 °C.

### Bacterial strains and media

*Escherichia coli* DH5α (New England Biolabs) was used for routine cloning and pathway propagation. Strains were maintained on Lysogeny broth (LB) or LB agar containing ampicillin (100 µg mL^−1^) for plasmid selection. Production strains are listed in Table 1.

**Table 1:** Bacterial strains used in this study

Strain name	Strain	Reference
DH5α (NEB 5-alpha)	K-12	New England Biolabs
DH10ß (NEB 10-beta)	K-12	New England Biolabs
DH1	K-12	Meselson & Yuan (1968)
MG1655	K-12	F. R. Blattner, et al. *Science* **277**, 1453–1462 (1997)
W3110	K-12	K. Hayashi, et al. *Mol. Syst. Biol*. **2** (2006)
DH1	K-12	M. Meselson & R. Yuan. *Nature* **217**, 1110–1114 (1968)
BW25113	K-12	Datsenko & Wanner (2000)
MDS42 Meta	K-12	Scarab Genomics
BL21(DE3)	B	New England Biolabs
Mach1	W	ThermoFisher

Knockout of the *fabF* gene was accomplished by standard lambda red recombineering^33^. Briefly, the kanamycin resistance cassette-disrupted *fabF* gene from the KEIO strain JW1081-4 (Δ*fabF759::kan*)^34^ was PCR amplified from genomic DNA using primers deltaFabF-F and deltaFabF-R to include ~250 bp sequences which flank the *fabF* gene in the *E. coli* genome. The MG1665 and MDS42 strains were transformed with the pSIM18 plasmid^35^, grown to an OD_600_ of 0.3 then heat shocked at 42 °C for 15 min to induce FLP recombinase expression, electrocompetent cells were prepared from these cultures. Aliquot of 50 µl of cells were transformed with 300 ng of the kanamycin cassette PCR product and then plated on agar plates containing 50 µg mL^−1^ kanamycin. Knockout of the *fabF* gene was confirmed by colony PCR using the primers deltaFabFCHK-F and deltaFabFCHK-R. Cells were cured of the pSIM18 plasmid by growth in liquid culture at 43 °C for 4–5 h. Loss of the pSIM18 cassette was then confirmed by replica plating of single colonies on LB agar plates containing 50 μg mL^−1^ kanamycin (growth) or 150 μg mL^−1^ hygromycin B (no growth).

### Automated robotic platforms

Plasmid libraries were screened in *E. coli* DH5α using an HTP 96-deep-well plate-based growth pipeline. Robotic platforms were implemented for automated colony picking, growth and induction with 24 h off-platform fermentation followed by automated product harvesting from the culture supernatant using methanol/water extraction. We assayed target compound titers from triplicate colonies using a 96-well plate HTP-LC-MS/MS (QqQ) quantitative system based on fast chromatography and automated MRM data extraction for selective Q1Q3 transitions for each compound.

Hamilton Star robotics platforms have been optimized for the efficient assembly of DNA pathways and the analysis of downstream combinatorial libraries. Equipped with both 8 channel and 96 head liquid handling, integrated PCR (TRobot), colony picking, growth and induction methods and metabolite extraction from cultured supernatant (Supplementary Fig. 1). Automated worklist packages generated from our Design software provide the complete recipes required for automated pathway assembly, coupled with integrated barcode reading for quality control and sample tracking.

### Part preparation

DNA parts (Supplementary Table 3) were synthesized by commercial vendors (Life Technologies, Germany; Gen9, USA; Twist Biosciences, USA) and amplified using part specific 5′-phosphorylated primers (Integrated DNA Technologies, Belgium; Supplementary Data 1). PCR reactions were treated with *Dpn*I (NEB), spin column-purified, analyzed by gel or capillary electrophoresis, quantified using a Nanodrop (ThermoFisher) and diluted to 75 nM with deionized water for ligase cycling reaction assembly. Parts were made fresh for each assembly and stored at 4 °C for <5 days.

### Plasmid assembly

An automated pipeline was created on the robotic platforms to reduce human error and ensure consistency and accuracy of assembly. Parts were produced by commercial DNA synthesis vendors followed by PCR amplification and processing. The pathways were built using the ligase cycling reaction assembly method^16,36^. Automated, worklist-driven liquid handling was implemented for bridging oligo pooling and ligase cycling reactions setup. Completed reactions were transformed into high efficiency NEB 5-alpha cells. Typically, 100–300 colonies were seen following overnight growth and five colonies of each were grown in 1.2 mL LB media for plasmid isolation using a QIAprep 96 Turbo Kit (Qiagen). Correct plasmid assembly was screened by automated restriction digests using *Eco*RI and *Bam*HI (unique restriction sites in all BglBrick plasmids^22^ to separate vector backbones from pathway inserts) followed by analysis on a 96-capillary Fragment Analyser (Advanced Analytical Technologies, USA). Correct assemblies (Supplementary Table 1) were then confirmed by full-pathway sequencing (GATC Biotech). Through this assembly pipeline we typically obtained >70% of sequence-perfect plasmid targets at the first attempt. We were successful in assembling the full 16-member pathway library in the first DBTL round (Fig. 2a) and 25 constructs for the second round 32-member pathway library (Fig. 2b).

### Pinocembrin production experiments

Production experiments were conducted in TBsb media (phosphate-buffered Terrific broth supplemented with 0.5 M sorbitol and 5 mM betaine) to approximate the conditions of Fehér et al.^21^. Additional media prepared for media screening (Table 2) were AIM (auto induction media, Formedium AIMTB0210), EZ (EZ-rich defined medium kit, Teknova M2105), MOPS (prepared as EZ but excluding 10× ACGU and 5× Supplement EZ), SOB (Super Optimal broth, Formedium SOB0202), TB (Terrific broth phosphate buffered, Formedium TBP0102) and M9 (recipe from Sambrook and Russell^37^). All media were supplemented with ampicillin (100 µg mL^−1^) and 0.4% glycerol (replacing glucose in the case of EZ media).

**Table 2:** Growth media used in this study

Designation	Name
EZ	EZ-rich defined medium
M9	M9 minimal medium
MOPS	MOPS minimal medium
SOB	Super optimal broth
TB	Terrific broth
TBsb	Terrific broth + 0.5 M sorbitol + 5 mM betaine

Overnight seed cultures were grown from freshly transformed colonies in the desired production media at 37 °C with shaking at 180 rpm. Seed cultures were inoculated 1/50 into fresh media (1.2 mL in 96-deep-well blocks with breathable seals) and grown at 30 °C, 950 rpm shaking, 75% humidity. When cultures reached OD_600_ = 1.2–1.5 (or OD_600_ = 0.5–0.6 for MOPS and M9 minimal media) they were induced with the addition of 100 µM IPTG, 3 mM phenylalanine and 20 µg mL^−1^ cerulenin, as appropriate, then transferred back to the 30 °C shaker-incubator for 24 h. Cultures were processed for analysis as described below. All liquid handling steps for plate-based experiments were performed on an Hamilton Star robotic platform.

### Reticuline/scoulerine production experiments

Production experiments were conducted in phosphate-buffered TB media supplemented with kanamycin (50 µg mL^−1^) and 0.4% glycerol. Overnight seed cultures were grown from freshly transformed colonies in production media at 37 °C with shaking at 180 rpm. Seed cultures were inoculated 1/50 into fresh media (1.2 mL in 96-deep-well blocks with breathable seals) and grown at 30 °C, 950 rpm shaking, 75% humidity. When cultures reached OD_600_ = 1.0 they were induced with the addition of 100 µM IPTG, returned to the shaker-incubator for 2 h, then pelleted by centrifugation at 2250 RCF for 10 min (5804R centrifuge; Eppendorf, Germany). Induced cells were resuspended in fresh production media supplemented with 500 µM THP substrate, 30 mM l-ascorbic acid and 100 µM IPTG, and transferred back to the 30 °C shaker-incubator for 24 h. Cultures were then processed for analysis as described below. All liquid handling steps for plate-based experiments were performed on an Hamilton Star robotic platform.

### Analysis and quantification of pathway targets

All cell cultures were quenched with an equal volume of 100% methanol at the 24 h time point post induction. The samples were then centrifuged at 2250 RCF for 10 min (5804R centrifuge; Eppendorf, Germany). The supernatant was collected and diluted as required with methanol/water (10:90 v/v) in 96-well plates. These plate-based extractions were designed to be compatible for analysis on a triple quadrupole tandem mass spectrometer (Xevo TQ-S; Waters MS Technologies) connected to an Acquity Ultra Performance Liquid Chromatography system (Acquity UPLC; H-Class, Waters) with a 96-well plate sample tray accessory. UPLC methods were optimized for the resolution of cinnamic acid and pinocembrin (flavonoids pathway) on a BEH C_18_ column (2.1 × 50 mm, 1.7 μm; Waters), and THP, reticuline and scoulerine (alkaloids pathway) on an HSS T3 column (2.1 × 50 mm, 1.8 μm; Waters). Mobile phase A (water + 0.05% formic acid) and mobile phase B (methanol + 0.05% formic acid) were used at an operating temperature of 45 °C (flavonoids pathway) or 40 °C (alkaloids pathway). Instrument injection sequences were randomly generated by Excel-based data tracking and worklist generators.

The optimized flavonoids LC gradient ran at a flow rate of 0.6 mL min^−1^, resulting in a total runtime of 2 min per sample. A linear gradient of 40–95% B (v/v) was applied over 1.5 min, before returning linearly to 40% B (v/v) over 1 min. A total of 40% B (v/v) was then maintained for 0.4 min to equilibrate the system for the next injection. The optimized alkaloids LC gradient ran at a flow rate of 0.6 mL min^−1^, resulting in a total runtime of 5 min per sample. A linear gradient of 1–63% B (v/v) was applied over 2.0 min, then increased to 95% B (v/v) over 0.1 min, before holding at 95% B (v/v) for 0.3 min. The gradient returned linearly to 1% B (v/v) over 0.1 min and was then maintained at 1% B (v/v) for 2.5 min to equilibrate the system for the next injection. The MS parameters were optimized with a desolvation gas flow of 1000 L h^−1^, a capillary voltage of 2000 V, desolvation temperature of 600 °C, and a source temperature of 150 °C. The MRM transition of 255.25 > 213.11 was used for the quantification of pinocembrin. MRM transitions of 288.15 > 164.17, 330.26 > 192.08, and 328.12 > 178.14 were used for quantification of THP, reticuline, and scoulerine, respectively.

The limit of detection (LOD) and limit of quantification (LOQ) were calculated from the signal-to-noise ratio (S/N). The LOD was defined when S/N > 3, and the LOQ defined when S/N > 10. Absolute quantification of targets was performed relative to an external 8-point calibration curve analyzed in the same analytical run as the samples. Relative comparison of cinnamic acid within the first two screening cycles were based on the cinnamic acid peak areas, which provided relative quantification and informed the DESIGN and BUILD for further pipeline optimization. A standard stock solution of (2*S*)-pinocembrin (4 mM) was freshly prepared in ethanol, then dilution series were prepared in 0.2% TB media in MeOH/H_2_O (10:90 v/v) to take into account matrix effects from the culture medium. Alkaloid standards were freshly prepared by adding THP (2 mM), (*S*)-reticuline (200 µM), and (*S*)-scoulerine (100 µM) to phosphate-buffered TB media containing 30 mM l-ascorbic acid, then dilution series were prepared in MeOH/H_2_O (10:90 v/v). The linear model for standard curves was selected based on the analysis of the data by linear regression with and without intercept and weighting factors 1/x. The linear range for pinocembrin quantification was 120 pM–500 nM. Linear ranges of 100 pM–400 nM, 10 pM–40 µM and 5 pM–20 nM were used for quantification of THP, reticuline, and scoulerine, respectively. MassLynx V4.1 SCN905, with TargetLynx (Waters Corp., Milford, MA, USA) was used to process the acquired data.

Binary MS/MS files were automatically transferred and archived with corresponding metadata using a data acquisition service. The binary MS/MS files were then transformed using Proteowizard^38^ to open-source mzXML format files from which we extracted peak areas from selective MRM transitions and performed absolute quantification in an automated manner. Custom in-house scripts used for peak areas extraction are available online at https://gitlab.cs.man.ac.uk/mqbpwca2/RP2.

### Statistical analysis

In order to analyze the significance of the effects for each factor, we performed a standard least squares analysis. Promoters were considered as a categorical factor, plasmid copy numbers were considered as an ordinal factor with two possible values: low and high copy number. *P* values corresponded to the probability associated with an F-test of zero effect of the parameter. All tests were carried out using JMP Pro 12.2.0 (SAS Institute).

Box-plot elements in figures: center line, median; box limits, upper and lower quartiles; whiskers, 1.5× interquartile range; points, outliers.

### Data management support

The integrated pipeline benefited from the support of a data management system consisting of commercial, open source, and bespoke data management platforms, which assisted in making the data FAIR (findable, accessible, interoperable, reusable)^39^ throughout the pipeline. An instance of the open-source JBEI Inventory of Composable Elements (ICE) registry^16,17^ was used as registry of parts, plasmids, and strains. Recording of experiments was performed using shared electronic lab notebooks (http://www.labarchives.com). A data acquisition service was developed in house, which allowed data to be remotely transferred from laboratory instruments (e.g., QqQ), archived and backed up in our large data store (Synology NAS Disk Station with mirrored backup). During transfer, data were simultaneously ingested into our OpenBis software^39^ along with associated metadata for easy retrieval.

### Data availability

The data sets generated during and/or analyzed during the current study are available in the Mendeley Data repository, 10.17632/8g4wfwtd43.1^40^. All synthetic DNA sequences designed and used in this study are publicly available from our ICE repository at https://ice.synbiochem.co.uk (see also Supplementary Table 1).

## Electronic supplementary material

Supplementary Information

Description of additional Supplementary Infomation

Supplementary Data 1

Supplementary Data 2

Supplementary Data 3

Supplementary Data 4

Supplementary Data 5

Supplementary Data 6

Supplementary Data 7

Supplementary Data 8
