# Supplementary material for: An automated Design-Build-Test-Learn pipeline for enhanced microbial production of fine chemicals
Source: Commun Biol. 2018 Jun 8;1:66. doi: 10.1038/s42003-018-0076-9 (PMC6123781; doi:10.1038/s42003-018-0076-9)
Supplement: Supplementary file 1 — Supplementary Information [file 42003_2018_76_MOESM1_ESM.pdf]

Supplementary Table 1. Plasmids used in the pinocembrin study.

**Plasmid reference**	**Description (replication origin – antibiotic resistance – pathway promoter/gene organization)**
pQlinkNHHHL	ColE1-AmpR-P_{T5}-AtPAL-P_{T5}-Sc4CL-P_{T5}-AtCHS-P_{T5}-AtlCHI*
SBC001040	p15A-AmpR-PlacUV5-AtlCHI-AtPAL-Ptrc-Sc4CL-PlacUV5-AtCHS**
SBC001041	p15A-AmpR-Ptrc-AtlCHI-AtPAL-Sc4CL-AtCHS
SBC001042	p15A-AmpR-PlacUV5-AtPAL-Sc4CL-PlacUV5-AtCHS-Ptrc-AtlCHI
SBC001043	p15A-AmpR-Ptrc-AtPAL-Sc4CL-AtCHS-AtlCHI
SBC001044	p15A-AmpR-PlacUV5-AtCHS-Ptrc-AtlCHI-AtPAL-Sc4CL
SBC001045	pSC101-AmpR-Ptrc-AtCHS-AtlCHI-Ptrc-AtPAL-Sc4CL
SBC001046	pSC101-AmpR-Ptrc-Sc4CL-AtCHS-PlacUV5-AtlCHI-AtPAL
SBC001047	pSC101-AmpR-PlacUV5-AtPAL-PlacUV5-Sc4CL-Ptrc-AtCHS-AtlCHI
SBC001048	pSC101-AmpR-PlacUV5-Sc4CL-AtCHS-AtlCHI-PlacUV5-AtPAL
SBC001049	p15A-AmpR-Ptrc-Sc4CL-Ptrc-AtCHS-Ptrc-AtlCHI-Ptrc-AtPAL
SBC001050	pSC101-AmpR-Ptrc-AtPAL-Ptrc-Sc4CL-AtCHS-PlacUV5-AtlCHI
SBC001051	pSC101-AmpR-PlacUV5-AtlCHI-Ptrc-AtPAL-PlacUV5-Sc4CL-AtCHS
SBC001052	p15A-AmpR-PlacUV5-Sc4CL-PlacUV5-AtCHS-AtlCHI-AtPAL
SBC001053	pSC101-AmpR-Ptrc-AtlCHI-PlacUV5-AtPAL-Sc4CL-Ptrc-AtCHS
SBC001054	p15A-AmpR-Ptrc-AtCHS-PlacUV5-AtlCHI-PlacUV5-AtPAL-PlacUV5-Sc4CL
SBC001055	pSC101-AmpR-PlacUV5-AtCHS-AtlCHI-AtPAL-Ptrc-Sc4CL

SBC003338	ColE1-AmpR-Ptrc-AtlCHI-Sc4CL-AtCHS-Ptrc-AtPAL
SBC003350	ColE1-AmpR-Ptrc-AtlCHI-Ptrc-Sc4CL-AtCHS-Ptrc-AtPAL
SBC003353	ColE1-AmpR-Ptrc-AtlCHI-Ptrc-Sc4CL-Ptrc-AtCHS-Ptrc-AtPAL
SBC003354	ColE1-AmpR-Ptrc-AtlCHI-Ptrc-Sc4CL-PlacUV5-AtCHS-Ptrc-AtPAL
SBC003358	ColE1-AmpR-Ptrc-AtlCHI-PlacUV5-Sc4CL-Ptrc-AtCHS-Ptrc-AtPAL
SBC003360	ColE1-AmpR-Ptrc-AtlCHI-AtCHS-Sc4CL-Ptrc-AtPAL
SBC003364	ColE1-AmpR-Ptrc-AtlCHI-AtCHS-Ptrc-Sc4CL-Ptrc-AtPAL
SBC003372	ColE1-AmpR-Ptrc-AtlCHI-Ptrc-AtCHS-Ptrc-Sc4CL-Ptrc-AtPAL
SBC003373	ColE1-AmpR-Ptrc-AtlCHI-Ptrc-AtCHS-PlacUV5-Sc4CL-Ptrc-AtPAL
SBC003376	ColE1-AmpR-Ptrc-AtlCHI-PlacUV5-AtCHS-Ptrc-Sc4CL-Ptrc-AtPAL
SBC003378	ColE1-AmpR-PlacUV5-AtlCHI-Sc4CL-AtCHS-Ptrc-AtPAL
SBC003379	ColE1-AmpR-PlacUV5-AtlCHI-Sc4CL-Ptrc-AtCHS-Ptrc-AtPAL
SBC003381	ColE1-AmpR-PlacUV5-AtlCHI-Ptrc-Sc4CL-AtCHS-Ptrc-AtPAL
SBC003382	ColE1-AmpR-PlacUV5-AtlCHI-Ptrc-Sc4CL-Ptrc-AtCHS-Ptrc-AtPAL
SBC003383	ColE1-AmpR-PlacUV5-AtlCHI-Ptrc-Sc4CL-PlacUV5-AtCHS-Ptrc-AtPAL
SBC003384	ColE1-AmpR-PlacUV5-AtlCHI-PlacUV5-Sc4CL-AtCHS-Ptrc-AtPAL
SBC003385	ColE1-AmpR-PlacUV5-AtlCHI-PlacUV5-Sc4CL-Ptrc-AtCHS-Ptrc-AtPAL
SBC003386	ColE1-AmpR-PlacUV5-AtlCHI-PlacUV5-Sc4CL-PlacUV5-AtCHS-Ptrc-AtPAL
SBC003387	ColE1-AmpR-PlacUV5-AtlCHI-AtCHS-Sc4CL-Ptrc-AtPAL

SBC003388	ColE1-AmpR-PlacUV5-AtlCHI-AtCHS-Ptrc-Sc4CL-Ptrc-AtPAL
SBC003389	ColE1-AmpR-PlacUV5-AtlCHI-AtCHS-PlacUV5-Sc4CL-Ptrc-AtPAL
SBC003391	ColE1-AmpR-PlacUV5-AtlCHI-Ptrc-AtCHS-Ptrc-Sc4CL-Ptrc-AtPAL
SBC003392	ColE1-AmpR-PlacUV5-AtlCHI-Ptrc-AtCHS-PlacUV5-Sc4CL-Ptrc-AtPAL
SBC003393	ColE1-AmpR-PlacUV5-AtlCHI-PlacUV5-AtCHS-Sc4CL-Ptrc-AtPAL
SBC003394	ColE1-AmpR-PlacUV5-AtlCHI-PlacUV5-AtCHS-Ptrc-Sc4CL-Ptrc-AtPAL

*Fehér *et al.*, 2014
**Plasmid DNA sequences are available as entries in the ICE repository, e.g. https://ice.synbiochem.co.uk/entry/SBC001040

Supplementary Table 2. Plasmids used in the reticuline/scoulerine study.

Plasmid reference	Description (replication origin – antibiotic resistance – pathway promoter/gene organization)
SBC000202	BBR1-KanR-Ptrc-Cj6OMT-Ptrc-CjCNMT-Cj4OMT-PlacUV5-CjBBE
SBC000203	ColE1-KanR-PlacUV5-Cj6OMT-CjCNMT-PlacUV5-Cj4OMT-Ptrc-CjBBE
SBC000204	BBR1-KanR-Ptrc-CjBBE-PlacUV5-Cj6OMT-CjCNMT-Ptrc-Cj4OMT
SBC000205	BBR1-KanR-PlacUV5-Cj6OMT-PlacUV5-CjCNMT-Ptrc-Cj4OMT-CjBBE
SBC000206	ColE1-KanR-Ptrc-CjCNMT-Ptrc-Cj4OMT-Ptrc-CjBBE-Ptrc-Cj6OMT
SBC000207	ColE1-KanR-PlacUV5-CjBBE-Cj6OMT-Ptrc-CjCNMT-PlacUV5-Cj4OMT
SBC000208	BBR1-KanR-PlacUV5-CjCNMT-Cj4OMT-CjBBE-PlacUV5-Cj6OMT
SBC000209	ColE1-KanR-Ptrc-Cj4OMT-PlacUV5-CjBBE-PlacUV5-Cj6OMT-PlacUV5-CjCNMT
SBC000210	BBR1-KanR-PlacUV5-Cj4OMT-CjBBE-Cj6OMT-Ptrc-CjCNMT
SBC000211	BBR1-KanR-Ptrc-CjCNMT-Cj4OMT-PlacUV5-CjBBE-Cj6OMT
SBC000212	ColE1-KanR-PlacUV5-Cj4OMT-Ptrc-CjBBE-Cj6OMT-CjCNMT
SBC000213	ColE1-KanR-Ptrc-CjBBE-Cj6OMT-CjCNMT-Cj4OMT
SBC000214	BBR1-KanR-PlacUV5-CjBBE-Ptrc-Cj6OMT-PlacUV5-CjCNMT-Cj4OMT

SBC000215	BBR1-KanR-Ptrc-Cj4OMT-CjBBE-Ptrc-Cj6OMT-CjCNMT
SBC000216	ColE1-KanR-PlacUV5-CjCNMT-PlacUV5-Cj4OMT-CjBBE-Cj6OMT
SBC000217	ColE1-KanR-Ptrc-Cj6OMT-CjCNMT-Cj4OMT-CjBBE

Supplementary Table 3. DNA parts list used in the pinocembrin study.

Part	Class	Description	Uniprot code
SBC000133	Vector backbone	pBbS1a	n/a
SBC000134	Vector backbone	pBbS5a	n/a
SBC000137	Vector backbone	pBbA1a	n/a
SBC000138	Vector backbone	pBbA5a	n/a
SBC000088	Terminator-promoter	B1006-Ptrc	n/a
SBC000124	Terminator-promoter	B1006-Ptrc	n/a
SBC000125	Terminator-promoter	B1006-Ptrc	n/a
SBC000126	Terminator-promoter	B1006-PlacUV5	n/a
SBC000127	Terminator-promoter	B1006-PlacUV5	n/a
SBC000128	Terminator-promoter	B1006-PlacUV5	n/a
SBC000096	Gene	AtPAL	P35510
SBC000099	Gene	Sc4CL	Q9K3W1
SBC000103	Gene	AtCHS	P13114
SBC000104	Gene	AtCHI	Q9FKW3

Supplementary Table 4. DNA parts list used in the reticuline/scoulerine study.

Part	Class	Description	Uniprot code
SBC000139	Vector backbone	pBbB1k	n/a
SBC000140	Vector backbone	pBbB5k	n/a
SBC000143	Vector backbone	pBbE1k	n/a
SBC000144	Vector backbone	pBbE5k	n/a
SBC000088	Terminator-promoter	B1006-Ptrc	n/a
SBC000124	Terminator-promoter	B1006-Ptrc	n/a
SBC000125	Terminator-promoter	B1006-Ptrc	n/a
SBC000126	Terminator-promoter	B1006-PlacUV5	n/a
SBC000127	Terminator-promoter	B1006-PlacUV5	n/a
SBC000128	Terminator-promoter	B1006-PlacUV5	n/a
SBC000120	Gene	Cj6OMT	Q9LEL6
SBC000121	Gene	CjCNMT	Q948P7
SBC000122	Gene	Cj4’OMT	Q9LEL5
SBC000112	Gene	CjBBE	K0IQX2

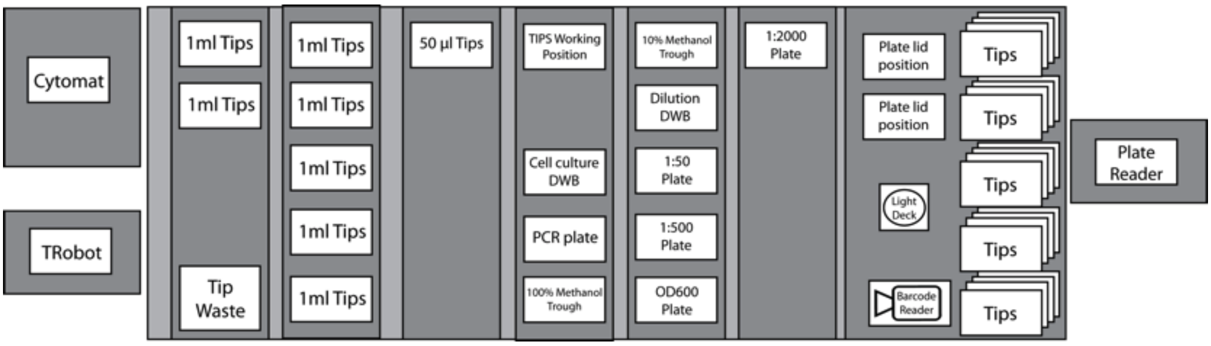

Supplementary Fig. 1. Hamilton Microlab STAR deck layout for automated extraction of samples for MS. Platform includes integrated Biometra TRobot (thermocycler), barcode reader, light deck for colony picking, CLARIOstar plate reader and temperature controlled cooling unit (Cytomat). All tip positions are standard 300µl nested racks unless stated. Labware positions include 3x destination sample plates 1:50, 1:500 and 1:2000, microtiter plate for OD_{600} reads, PCR plate for initial quenching of reactions, deep-well block for sample dilution and 2x troughs containing 100% and 10% methanol. Briefly, an initial OD_{600} measurement is taken using an integrated CLARIOstar plate reader before cells are quenched in 100% methanol. Samples are vortexed and centrifuged (off line) before diluting in 10% methanol up to a final ratio of 1:2000. Plates are sealed and run on a HTP-LC-MS/MS (QqQ) quantitative system.
