# Supplementary material for: An automated Design-Build-Test-Learn pipeline for enhanced microbial production of fine chemicals
Source: Commun Biol. 2018 Jun 8;1:66. doi: 10.1038/s42003-018-0076-9 (PMC6123781; doi:10.1038/s42003-018-0076-9)
Supplement: Supplementary file 2 — Description of additional Supplementary Infomation [file 42003_2018_76_MOESM2_ESM.pdf]

Supplementary Data

Supplementary Data 1. Oligonucleotides used in the pinocembrin study.

Supplementary Data 2. Oligonucleotides used in the reticuline/scoulerine study.

Supplementary Data 3. Quantified pinocembrin titers and cinnamic acid peak values (mean, standard deviation, standard error of the mean, median, interquartile range), and mean OD_{600} values at induction and harvest points, for the combinatorial plasmid library of the first DBTL round. The library was generated through a DoE approach by varying promoters, gene arrangement and plasmid copy number. Plasmids were assembled by ligase cycling reaction, transformed into *E. coli* DH5α, then grown in triplicate in TBsb media (0.4% glycerol) at 30 °C. Cultures were quenched and processed for analysis 24 h after pathway induction by IPTG.

Supplementary Data 4. Quantified pinocembrin titers and cinnamic acid peak values (mean, standard deviation, standard error of the mean, median, interquartile range), and mean OD_{600} values at induction and harvest points, for the combinatorial plasmid library of the second DBTL round. The library is a full factorial focused library for selected factors that were identified as having the highest effect on pinocembrin titers in the first DBTL round. Plasmids were assembled by ligase cycling reaction, transformed into *E. coli* DH5α, then grown in triplicate in TBsb media (0.4% glycerol) at 30 °C. Cultures were quenched and processed for analysis 24 h after pathway induction by IPTG.

Supplementary Data 5. Quantified pinocembrin titers and cinnamic acid peak values (mean, standard deviation, standard error of the mean, median, interquartile range), and mean OD_{600} values at induction and harvest points, for chassis selection experiments. The three best performing constructs from the second DBTL cycle (plasmids 3353, 3382, and 3391) were screened in triplicate in a library of *E. coli* strains (Table 1) grown at 30 °C in TBsb media supplemented with 0.4% glycerol. Cultures were quenched and processed for analysis 24 h after pathway induction by IPTG.

Supplementary Data 6. Quantified pinocembrin and cinnamic acid titers (mean, standard deviation, standard error of the mean, median, interquartile range), and mean OD_{600} values at induction and harvest points, for media selection experiments. The best performing construct (plasmid 3382) was screened in quadruplicate in *E. coli* MG1655 and MDS42 strains grown at 30 °C in the indicated

media supplemented with 0.4% glycerol. Cultures were quenched and processed for analysis 24 h after pathway induction by IPTG.

Supplementary Data 7. Quantified pinocembrin titers and cinnamic acid titers (mean, standard deviation, standard error of the mean, median, interquartile range), and mean OD_{600} values at induction and harvest points, for titer optimization experiments. The chromosomal *fabF* gene was knocked out in *E. coli* MG1655 and MDS42, then these strains were transformed with the best performing construct (plasmid 3382). To elucidate the optimal induction point for pinocembrin production, cultures were screened in quadruplicate following induction at different OD_{600} values (Odi). Cultures were quenched and processed for analysis 24 h after pathway induction by IPTG.

Supplementary Data 8. Quantified reticuline and scoulerine titers (mean, standard deviation, standard error of the mean, median, interquartile range), and mean OD_{600} values at induction and harvest points, for the combinatorial plasmid library. The library was generated through a DoE approach by varying promoters, gene arrangement and plasmid copy number. Plasmids were assembled by ligase cycling reaction, transformed into *E. coli* DH5α, then grown in triplicate in phosphate-buffered TB media (0.4% glycerol) at 30 °C. Cultures were quenched and processed for analysis 24 h after THP substrate was added to IPTG induced cells.
